# Reducing HuD Levels Alleviates Alzheimer's Disease Pathology in 5xFAD Mice

**DOI:** 10.1111/acel.70080

**Published:** 2025-05-12

**Authors:** Eunbyul Ji, Apala Pal, Quia C. Claybourne, Marc Michel, Rachel Munk, Ross A. McDevitt, Chang‐Yi Cui, Myriam Gorospe

**Affiliations:** ^1^ Laboratory of Genetics and Genomics, National Institute on Aging (NIA) Intramural Research Program (IRP), National Institutes of Health (NIH) Baltimore Maryland USA; ^2^ Comparative Medicine Section National Institute on Aging (NIA) Intramural Research Program, National Institutes of Health (NIH) Baltimore Maryland USA

**Keywords:** 5xFAD, Alzheimer's disease, aβ plaques, *Elavl4*, homecage activity, HuD

## Abstract

Alzheimer's disease (AD) is the most common neurodegenerative pathology in older persons. The accumulation of amyloid β (Aβ) plaques is a major contributor to AD development. The RNA‐binding protein HuD/ELAVL4 has been implicated in the formation of Aβ plaques, but its role in AD is unclear. Here, we report that ablation of *HuD* from CAMK2A^+^ neurons (HuDcKO) in the 5xFAD mouse model of AD results in a significant reduction of Aβ plaques and the alleviation of some AD‐associated behaviors. Given the lack of effective therapies for AD, we propose that reducing HuD levels or function can contribute to diminishing Aβ plaque formation and AD‐associated pathology.

## Graphical abstract

The effect of ablating expression of the RNA‐binding protein HuD (ELAVL4) in neurons was evaluated in the Alzheimer's mouse model 5xFAD. We found that conditional removal of HuD resulted in diminished Aβ plaque formation and reduced AD‐associated hyperactivity in 5xFAD/HuDcKO mice relative to 5xFAD mice.

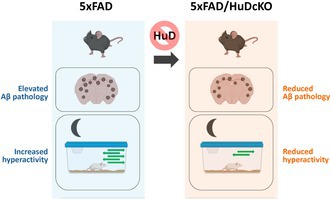

**Abbreviations**
AD: Alzheimer's disease
APP: amyloid precursor protein
Aβ: amyloid‐beta
cKO: conditional knockout
*Elavl4*
: embryonic lethal abnormal vision‐like 4
gDNA: genomic DNA
IHC: immunohistochemistry

## 1 Introduction

Alzheimer's disease (AD) is the most common neurodegenerative disorder and its main risk factor is age (Querfurth and LaFerla 2010; Guerreiro and Bras 2015). A major hallmark of AD pathology is the accumulation of amyloid beta (Aβ) plaques (Chen et al. 2017) and tau (MAPT) neurofibrillary tangles (Guo et al. 2017). These neurotoxic protein aggregates cause blood–brain barrier (BBB) impairment, neuroinflammation, mitochondrial dysfunction, and oxidative stress. In turn, these alterations cause irreversible brain damage, resulting in brain shrinkage, memory loss, and declining cognitive ability (Kent et al. 2020; Blinkouskaya and Weickenmeier 2021).

The RNA‐binding protein (RBP) HuD was previously found to bind mRNAs encoding the precursor of Aβ (the protein APP) and the protease BACE1, enhancing their expression and promoting the amyloidogenic cleavage of APP to release Aβ (Kang et al. 2014). A member of the embryonic lethal abnormal vision‐like (ELAVL)/Hu family (which also comprises HuA/HuR, HuB, and HuC), HuD is abundantly expressed in the brain, gonads, and pancreas (Jung and Lee 2021). In the brain, HuD plays a key role in neuronal development, differentiation, and function by modulating mRNA splicing, transport, stability, and translation (Deschênes‐Furry et al. 2006; Hinman and Lou 2008).

Although HuD has been associated with Aβ plaque formation in human and mouse (Subhadra et al. 2013; Kang et al. 2014; van der Linden et al. 2022), other studies suggested an opposite role, reporting a decline of HuD with advancing AD (Amadio et al. 2009), and reporting that HuD mitigated AD‐related molecular changes in neurons derived from human induced pluripotent stem cell (iPSC) lines (van der Linden et al. 2022). Given these contrasting findings, we aimed to clarify the contribution of HuD to Aβ formation and AD pathogenesis in vivo. To this end, we generated a neuron‐specific *HuD* gene knockout (HuDcKO) in the AD mouse model 5xFAD, which expresses the human *APP* gene bearing three familial AD (FAD) mutations (KM670/671NL, V717I, I716V) and the human *PSEN1* gene bearing two FAD mutations (M146L and L286V) (Oakley et al. 2006). We report that Aβ plaque formation is significantly reduced and a typical AD phenotype, hyperactivity (Jul et al. 2016; Oblak et al. 2021), is fully reversed in the 5xFAD/HuDcKO mice. Our results underscore the value of strategies aimed at reducing HuD as a way to reduce AD‐associated pathology.

## 2 Results

**FIGURE 1:**
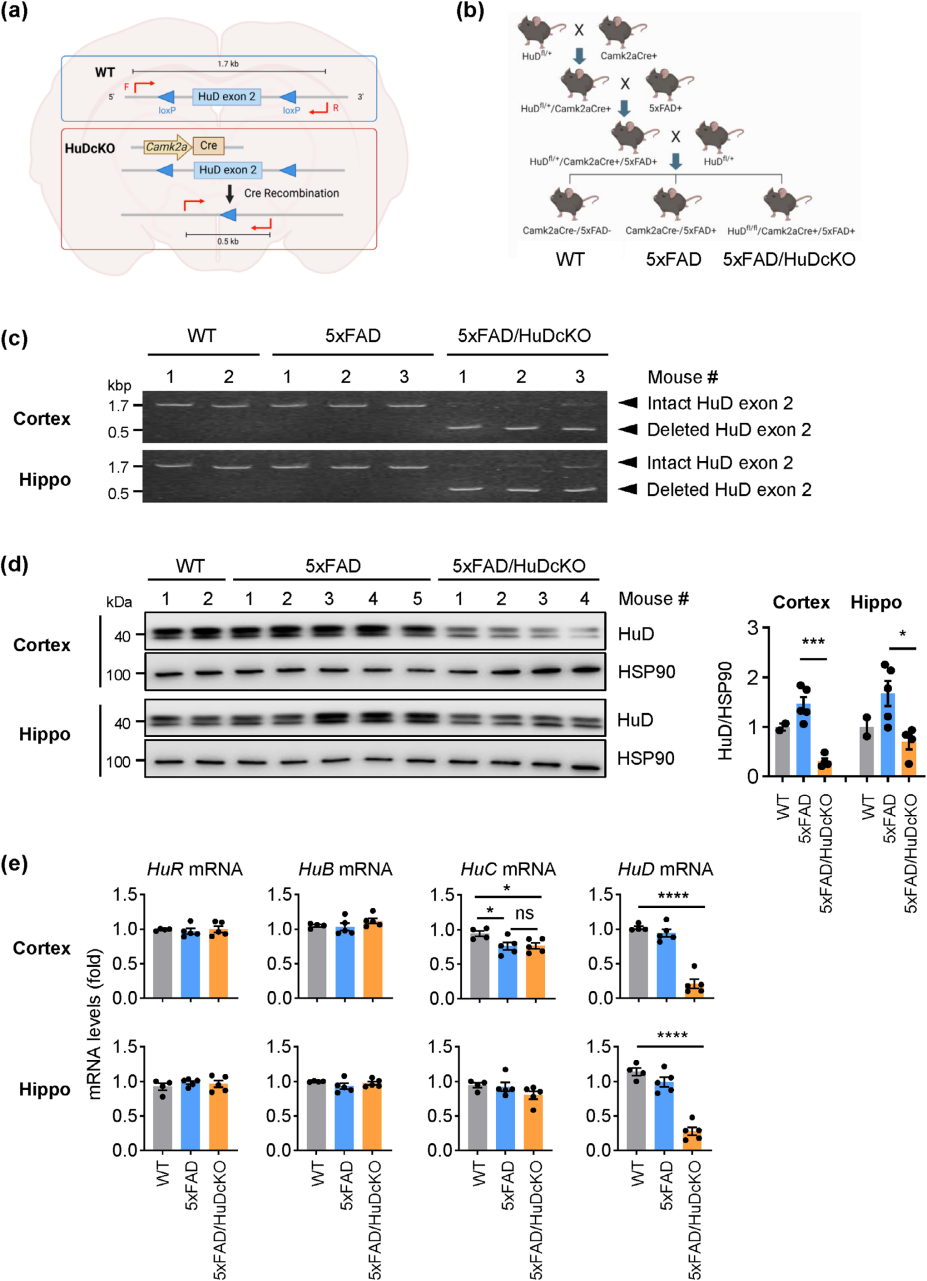
Generation and characterization of 5xFAD/HuDcKO mice. (a) Strategy to ablate the *HuD*/*Elavl4* gene by the loxP‐Cre system. Exon 2 of the HuD gene, flanked by two loxP sites, was designed to be excised by Cre recombinase under the control of the *Camk2a* promoter. Red arrows indicate the primers amplifying the floxed *HuD* exon 2 for genotyping. (b) Breeding scheme to generate HuDcKO mice in the 5xFAD background. (c) Recombination at the *HuD* locus was confirmed by PCR amplification of genomic DNA isolated from cerebral cortex (Cortex) and hippocampus (Hippo) of 9‐month‐old (9 m.o.) female mice using the primers indicated in panel a (red). (d) The levels of HuD protein from 9 m.o. female mice were assessed by western blot analysis using HSP90 as a loading control. Signal intensities were quantified using ImageJ. The numbers above the lanes in (c, d) indicate individual mice in each genotype group (biological replicates). (e) RNA was isolated from 9 m.o. female mice, and the levels of mRNAs encoding proteins in the Hu family and *Gapdh* mRNA (used for normalization) were measured by RT‐qPCR analysis. Data in (d, e) represent the means ± SEM. Statistical significance (* *p* < 0.05; ***p < 0.001; ****p < 0.0001) was assessed with Student's *t*‐test.

To study the effect of HuD on AD, we first generated an *HuD* mutant mouse model with a floxed exon 2 and crossed it with *Camk2a*‐Cre mice to generate neuron‐specific HuD mice with ablated exon 2 (HuDcKO) [Methods, Section 4, (Figure 1a)]. We then crossed the HuDcKO mice with 5xFAD mice (Oakley et al. 2006) to generate HuDcKO mice in an AD background (5xFAD/HuDcKO) (Figure 1b). We confirmed that *HuD* exon 2 was successfully ablated in the brain by genomic (g)DNA PCR analysis of cerebral cortex (Cortex) and hippocampus (Hippo); the region containing *HuD* exon 2 was amplified using the indicated primer set (red arrows, Figure 1a). WT and 5xFAD mice showed a single band at ~1.7 kbp, indicating an intact *HuD* exon 2 (Figure 1c). By contrast, 5xFAD/HuDcKO mice showed a major band at ~0.5 kbp and a faint band at ~1.7 kbp, indicating that *HuD* exon 2 was largely deleted in these brain regions, but deletion was incomplete in a small subset of cells. Consistent with the gDNA result, western blot analysis revealed that HuD protein expression levels were significantly lower in both brain regions of 5xFAD/HuDcKO mice relative to 5xFAD (Figure 1d); of note, the reduction in HuD levels was more pronounced in cortex than in hippocampus.

To confirm that *HuD* mRNA was reduced following the *Camk2a*‐Cre‐mediated ablation of *HuD* exon 2, and to test for possible compensatory increases in other Hu RBPs, the levels of mRNAs encoding the other Hu proteins were further measured in cortex and hippocampus by RT‐qPCR analysis. The abundance of *HuR* mRNA or *HuB* mRNA did not change in the mutant mouse brains, while the levels of *HuC* mRNA declined slightly in the cortex of 5xFAD mice, likely a consequence of AD‐associated neuronal loss in the 5xFAD mice (Ogawa et al. 2018; Salvadores et al. 2020) (Figure 1e). Overall, the knockout efficiency in the brain of this conditional mouse model is ~60%–90%, as determined by genotyping, western blot, and RT‐qPCR analyses (Figure 1c–e). The residual expression of HuD in the HuDcKO mice suggests that recombination in some *Camk2a*‐positive cells was incomplete or that some *Camk2a*‐negative cells may express HuD in the brain.

**FIGURE 2:**
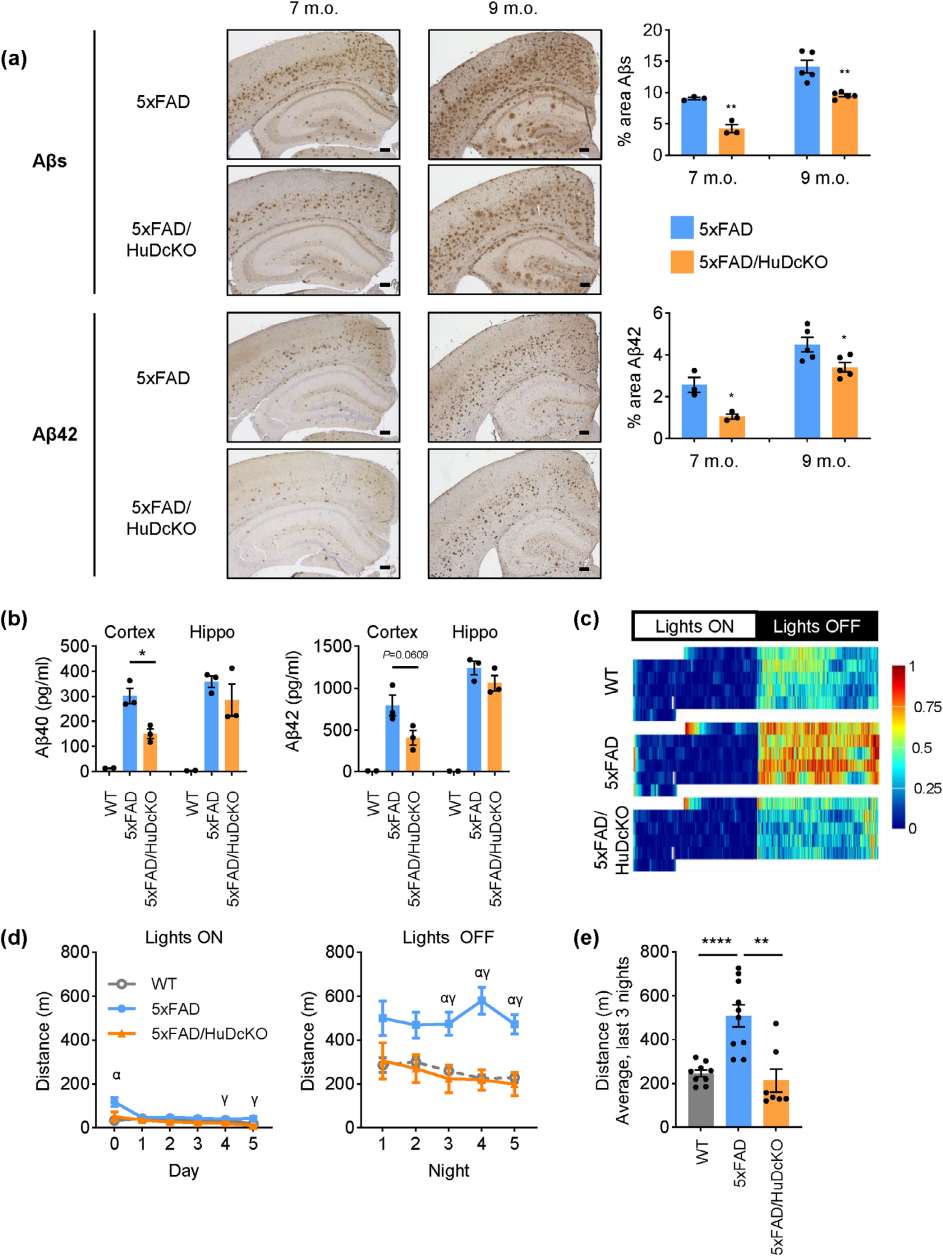
HuDcKO inhibits AD traits in 5xFAD. (a) The presence of plaques containing Aβ was analyzed by IHC with antibodies detecting Aβ peptides ranging in length between 37 and 42 kDa (Aβs, top) or specifically detecting Aβ42 (bottom) in 7 m.o. and 9 m.o. female mice. Aβ plaque signals were quantified as % area using ImageJ (*right*). Scale bars, 200 μm. (b) The concentration of Aβ40 and Aβ42 was measured in brain lysate from 7 m.o. female WT (*n* = 2), 5xFAD (*n* = 3), and 5xFAD/HuDcKO (*n* = 3) using BioPlex. (c–e) Homecage activity test was performed with 9 m.o. female mice that were WT (*n* = 9), 5xFAD (*n* = 10), or 5xFAD/HuDcKO (*n* = 7). (c) Daily homecage activity, shown in heatmap actograms, represents the distance traveled (m) per minute; the indents in days 0 and 5 reflect the fact that the recordings began and ended in mid‐morning, respectively. (d) Distance traveled in homecage was recorded during 12‐h light or dark phases across five nights. (e) Average distance traveled per night in the last three nights of testing, after mice had acclimated to the new surroundings. Data in (a,b,d,e) represent the means ± SEM. Statistical significance (*p < 0.05; **p < 0.01; ****p < 0.0001) was assessed using Student's *t*‐test or Tukey's test. Greek letters denote significant Tukey's pairwise comparisons between genotypes: α, WT versus 5xFAD; β, WT versus 5xFAD/HuDcKO; γ, 5xFAD versus 5xFAD/HuDcKO.

Aβ plaque formation in 5xFAD and 5xFAD/HuDcKO mice was evaluated in the cortex and hippocampus of mice aged 7 and 9 months old (m.o.) by immunohistochemical analysis to detect Aβ fragments (ranging from 37 to 42 kDa) or specifically Aβ42 (Figure 2a, left); the % area of stained Aβ plaques was quantified (Figure 2a, right). Importantly, in both age groups, Aβ plaques were significantly smaller in 5xFAD/HuDcKO mice compared to sex‐ and age‐matched 5xFAD mice, although the reduction in Aβ plaque formation appeared more effective at 7 m.o. than at 9 m.o. The concentrations of Aβ40 and Aβ42 in brain lysates of 7 m.o. mice were measured using a Bio‐Plex 200 System; as shown, strong reductions in Aβ40 and Aβ42 levels were quantified in 5xFAD/HuDcKO mice (Figure 2b).

In AD mouse models, the accumulation of Aβ plaques is linked to AD‐associated psychopathological behaviors, such as hyperactivity. Analysis of 9 m.o. mice singly housed in homecage monitoring chambers revealed that in the light cycle (‘Lights ON’, Figure 2c–e), the different genotypes behaved similarly. However, during the dark cycle (‘Lights OFF’, Figure 2c–e), 5xFAD mice were more active than WT mice, as anticipated (Oblak et al. 2021; Wang et al. 2024); importantly, 5xFAD/HuDcKO mice showed a strong amelioration of the 5xFAD phenotype, with behavior similar to WT. Quantification of the last 3 days of dark phase activity, when mice were acclimated to the chambers (Figure 2e), confirmed the result that hyperactivity in 5xFAD/HuDcKO mice was far lower than in 5xFAD mice, and was comparable to that of WT mice. These results support the view that HuDcKO reversed this trait of the 5xFAD mice. Collectively, our study indicates that reducing HuD levels in the 5xFAD brain alleviates Aβ plaque formation and a behavior (hyperactivity) often associated with AD.

## 3 Discussion

The current study addressed the impact of lowering HuD abundance in a prominent mouse model of AD, created by crossing the 5xFAD mouse with a mouse in which HuD was knocked out (strongly reduced) in neurons, with deletion of HuD driven by *Camk2a*‐Cre. Our results indicate that HuD production is an aggravating factor in AD, as AD‐associated histopathology (Aβ plaques in brain) and particularly AD‐associated hyperactive night‐time behavior were reduced after lowering HuD expression in the 5xFAD mouse.

Several reports have suggested a link between HuD and AD pathology. HuD abundance was found elevated in human AD brain (frontal cortex and superior temporal gyrus) compared to age‐matched non‐AD controls (Kang et al. 2014; Subhadra et al. 2013). HuD was reported to associate with *SERPINI1* mRNA and increased its stability (Bolognani and Perrone‐Bizzozero 2008); given that SERPINI1 (neuroserpin) is an inhibitor of tissue plasminogen activator (tPA), the authors proposed that the normal clearance of Aβ plaques by tPA is suppressed in AD brain by the rise in HuD levels (Subhadra et al. 2013). In cultured human neuroblastoma cells, HuD associated with *APP* and *BACE1* mRNAs, increased their stability, and elevated APP and BACE1 production, resulting in a rise in Aβ levels (Kang et al. 2014).

However, the connection between HuD levels and AD is complex. HuD and other neuronal Hu proteins (nELAV) bind and stabilize *ADAM10* mRNA, increasing production of an APP‐cleaving enzyme, the α‐secretase ADAM10 (which promotes a non‐amyloidogenic pathway). In the hippocampus of the AD brain, reduced levels of nELAV proteins were linked to decreased production of ADAM10, and hence a rise in Aβ (Pascale et al. 2005; Amadio et al. 2009). Additionally, overexpressing and reducing HuD expression in human iPSCs differentiated into neurons suggested that HuD in cultured cells ameliorated AD‐related molecular changes, including reductions in Aβ42/Aβ40 ratios (van der Linden et al. 2022). The contrasting results reported here, with loss of HuD reducing AD‐associated pathology, likely stem from variations in experimental design, including differences between studying mouse brain and cultured human cells, as well as differences in time elapsed since HuD was silenced or ablated. Although our data using a conditional HuD knockout mouse strongly support a role for HuD in exacerbating AD pathology, a deeper examination of the influence of HuD in AD pathology in this model is necessary. For example, it will be critical to study how cells surrounding neurons influence HuD function in this paradigm, and the consequences of modulating HuD levels in cells that express AD‐relevant mutations in different genes.

Whole‐body HuD knockout mice showed defects of neuronal development and motor function (Akamatsu et al. 2005) as well as abnormal behavior (DeBoer et al. 2014). However, we did not observe significant differences between WT and the HuDcKO mice we generated (data not shown). Although 5xFAD mice were smaller than WT mice, the body weights of 5xFAD/HuDcKO mice were comparable to those of 5xFAD mice (Figure S1a,b), highlighting minimum effects of our HuDcKO on the whole body. These differences may be related to the timing of the shutting off of HuD production, as HuD is critical for neuronal development and declines after birth (DeBoer et al. 2014). Alternatively, HuD is likely expressed in some CAMK2A‐negative cells in the brain, as suggested in our HuDcKO mice (Figure 1c–e), which may also contribute to the phenotypic differences between whole‐body HuD KO mice and our HuDcKO mice. Given that prenatal HuD deficiency led to severe developmental defects (Akamatsu et al. 2005; DeBoer et al. 2014), we minimized the confounding effects by using the *Camk2a*‐Cre recombination system, as the *Camk2a* promoter becomes active postnatally in neurons (Dragatsis and Zeitlin 2000).

Wandering and agitation are common among AD patients, and potentially analogous hyperactivity behavior is observed in several mouse models of AD (Holtzer et al. 2003; Ambrée et al. 2006; Oblak et al. 2021). Given that female 5xFAD mice are more susceptible to Aβ deposition and show greater hyperactivity than male 5xFAD mice (Sil et al. 2022; Lansdell et al. 2023), we primarily analyzed female mice, supplementing our analysis with available male mice for behavioral tests. Lowering HuD levels in the 5xFAD mice (5xFAD/HuDcKO) robustly reversed the hyperactivity phenotype seen in the 5xFAD mouse during the dark cycle, as tested in 9 m.o. female (Figure 2c–e) and 15 m.o. male mice (Figure S1c–e); other behavior tests did not show the same reversal (not shown).

In sum, reducing HuD in AD mice reduces Aβ accumulation and alleviates behavioral symptoms. As the urgent search for interventions in AD progresses, we propose that interventions to reduce HuD levels could be beneficial in therapies to delay or ameliorate AD.

## 4 Methods

Please see Supporting Information.

## Author Contributions

E.J., C.‐Y.C., and M.G. wrote the manuscript. E.J., C.‐Y.C., Q.C.C., and R.A.M. designed experiments and collected and analyzed data. A.P. and R.M. assisted with experiments. M.M. and C.‐Y.C. maintained the mouse colony. All authors contributed critical feedback that shaped the study and manuscript.

## Conflicts of Interest

The authors declare no conflicts of interest.

## Supporting information


Data S1.

**Table S1.** Details of PCR primers, antibodies, and other materials used in this study. The oligomers listed were used as primers to amplify genomic (g)DNA and reverse‐transcribed (RT) RNA, by quantitative (q)PCR analysis; forward and reverse primers for each amplicon are indicated. For the antibodies used in this study, relevant details (company, catalog number, and dilutions for detection) are indicated. The reagents, commercial kits, and mice used in this investigation, including companies and catalog numbers, are indicated.

## Data Availability

All data used to generate the statistical analyses and figures in this study are available from the corresponding author upon reasonable request.
