# Supplementary material for: Reducing HuD Levels Alleviates Alzheimer's Disease Pathology in 5xFAD Mice
Source: Aging Cell. 2025 May 12;24(6):e70080. doi: 10.1111/acel.70080 (PMC12151878; doi:10.1111/acel.70080)
Supplement: Supplementary file 1 — Data S1. [file ACEL-24-e70080-s001.pdf]

Supplementary Material

METHODS

Mice and behavior tests

All mouse work was done under an Animal Study Proposal (ASP # 476-LGG-2027), reviewed and approved by the Animal Care and Use Committee of the National Institute on Aging (NIA), National Institutes of Health (NIH). C57BL/6J, 5xFAD, and *Camk2a*-Cre mice were purchased from The Jackson Laboratory and floxed HuD mouse was custom-made by Cyagen. The 5xFAD strain B6.Cg-Tg(APPSwF1Lon,PSEN*M146L*L286V)6799Vas/Mmjax, RRID:MMRRC_03848-JAX (Oakley et al.., 2006), was obtained from the Mutant Mouse Resource and Research Center (MMRRC) at The Jackson Laboratory, an NIH-funded strain repository, and was donated to the MMRRC by Robert Vassar, Ph.D. (Northwestern University). Female mice at 7 m.o. (n=2 for WT; n=3 for 5xFAD and 5xFAD/HuDcKO, respectively) and at 9 m.o. (n=5 for WT; n=7 for 5xFAD and 5xFAD/HuDcKO, respectively) were euthanized by cervical dislocation. Brain hemispheres were directly fixed in 10% formalin in 1× PBS for IHC; cerebral cortex and hippocampus were extracted from the remaining hemispheres, frozen in liquid nitrogen, and stored at -80° C for molecular analysis.

Nineteen 9 m.o. female mice (n=10 for WT, n=9 for 5xFAD, and n=7 for 5xFAD/HuDcKO) and eighteen 15 m.o. male mice (n=8 for WT, n=5 for 5xFAD, and n=5 for 5xFAD/HuDcKO) were subjected to behavioral tests. Mice tested for behavior were singly housed for four nights in a Digital Ventilated Caging system (Tecniplast) with capacitive sensors beneath each cage to monitor activity.

Protein analysis

Tissues were homogenized in RIPA buffer [10 mM Tris-HCl (pH 7.5), 150 mM NaCl, 1 mM EDTA, 1% NP-40, 0.1% SDS, 0.5% sodium deoxycholate] containing 1× protease inhibitor cocktail (Roche), RNase inhibitor using the Precellys Lysing kit (Bertin Technologies) and Precellys® Tissue Homogenizer (Bertin Technologies), and the cleared lysates were collected after centrifugation for 15 min at 13,000 × g. Protein concentration was measured using the Bradford assay (Bio-Rad). Protein samples containing 1× SDS Laemmli sample buffer (Bio-Rad) were boiled at 95 °C for 15 min before loading, separated by electrophoresis through 4-20% SDS-polyacrylamide gels, and transferred onto nitrocellulose membrane using Trans-Blot Turbo transfer system with Trans-Blot Turbo RTA Midi 0.2 μm Nitrocellulose Transfer Kit (Bio-Rad). Membranes were blocked with 5%

non-fat milk for 1 h at room temperature and incubated with primary antibodies for 18 h at 4 °C. After washing with 1× TBS-T, the membranes were incubated with secondary antibodies for 30 min at room temperature. Following additional washing, membranes were briefly incubated with KwikQuant Western blot detection kit (Kindle Biosciences) and chemiluminescent signals on membranes were detected using ChemiDoc system (Bio-Rad). Primary antibodies used for western blot analysis are listed (Supplementary material, Table S1). The levels of HuD on western blots were quantified by densitometry analysis of HuD signals using the software ImageJ and normalized to the intensities of HSP90 signals.

gDNA analysis

Cortex and hippocampus (25 mg) were cut into small pieces, and gDNAs were isolated using DNeasy Blood & Tissue kit (Qiagen) following the manufacturer instructions; 200 ng gDNAs were used as templates and the regions containing floxed HuD exon 2 were amplified by PCR using exon2 primer set (Supplementary material, Table S1) with DreamTaq DNA polymerase (Thermo Scientific). PCR products were size-separated using E-gel EX 2% Agarose (Invitrogen) and captured using E-gel Power Snap Electrophoresis Device & Camera (Invitrogen) for further analysis.

mRNA analysis

Tissue lysates (100 µl) were mixed with the TriPure™ Isolation Reagent (Sigma-Aldrich) and RNA was isolated using Direct-zol RNA MiniPrep kit (Zymo Research) following the manufacturer instructions. Total RNA (1 µg) was subjected to reverse transcription (RT) using the Maxima Reverse Transcriptase (Thermo Fisher) with random hexamers, and transcript abundance was assessed by quantitative PCR (qPCR) analysis using SYBR green master mix (Kapa Biosystems) and gene-specific primer sets (Supplementary material, Table S1). RT-qPCR analysis was conducted using a QuantStudio 5 Real-Time PCR System (Thermo Fisher); RNA levels were quantified by the $2^{-\Delta\Delta Ct}$ method and normalized to *Gapdh* mRNA levels.

Immunohistochemistry (IHC)

Formalin-fixed brain hemispheres were trimmed, dehydrated, and embedded in paraffin. Paraffin-embedded tissue blocks were sectioned at 5-µm thickness using a microtome (Epredia HM 340 rotary microtome) and the slides were deparaffinized and rehydrated in a series of xylene, ethanol, and distilled water before staining. The tissue slides were then subjected to heat-induced antigen retrieval using 100× citrate buffer pH 6.0 (Abcam) and washed with 1× TBS-T. Aβ plaques on the

pre-treated tissue slides were stained with DAB using two anti-Aβ antibodies (Supplementary material, Table S1) and mouse- and rabbit-specific HRP/DAB (ABC) detection IHC kits (Abcam), following the manufacturer protocol. Sections were sequentially rinsed with 1× TBS-T, counterstained with hematoxylin, washed with tap water, dehydrated with ethanol and toluene, and mounted with organic mounting medium (Abcam). The images of stained Aβ plaques were captured by a microscope (Keyence) with 4× magnification, and % area was quantified using ImageJ.

Bioplex

Whole-cell lysates of murine cortex and hippocampus were centrifuged at 16,000×g, 4 ºC for 4 min, and Aβ40 and Aβ42 fragments in supernatants were measured using the Amyloid Beta Magnetic Bead Panel (Sigma Millipore #MABMAG-83K) following the manufacturer instructions. The final results were calculated using a Bio-Rad Bio-Plex® Automated 200 System with high throughput fluidics (HTF) and represented as pg/ml.

Statistical analysis

Data were expressed as the means ± SEM of three or more independent experiments, except for behavior testing, which was all done at once as one single experiment. Statistical significance was established by Student's t-test or ANOVA / repeated measures ANOVA followed by Tukey's using the GraphPad Prism 9.0 software; *, $p<0.05$; **, $p<0.01$; ***, $p<0.001$; ****, $p<0.0001$ and by Tukey's comparison between genotypes using the GraphPad Prism 9.0 software; α, WT *vs* 5xFAD; β, WT *vs* 5xFAD/HuDcKO; γ, 5xFAD *vs* 5xFAD/HuDcKO.

Supplementary Figure Legend

FIGURE S1 | Extended data, HuDcKO suppresses AD traits in the 5xFAD mouse

(a,b) 9-month-old (9 m.o.) female (a) and 15 m.o. male (b) mice were weighed before sacrifice (n=5 for WT, 5xFAD, and 5xFAD/HuDcKO). (c-e) For 15 m.o. male mice, the distance traveled in homecage during 12-hour light (c) and 12-hour dark (d) phases were recorded across five nights, and the average distance traveled per night in the last three nights of testing were calculated after the mice had acclimated to new caging and room (e). Data represent the means ± SEM. Statistical significance (*, $p < 0.05$; **, $p < 0.01$) was assessed with Student's t-test. Greek letters denote significant Tukey's pairwise comparisons between genotypes: α, WT vs 5XFAD; β, WT vs 5xFAD/HuDcKO; γ, 5xFAD vs 5xFAD/HuDcKO.

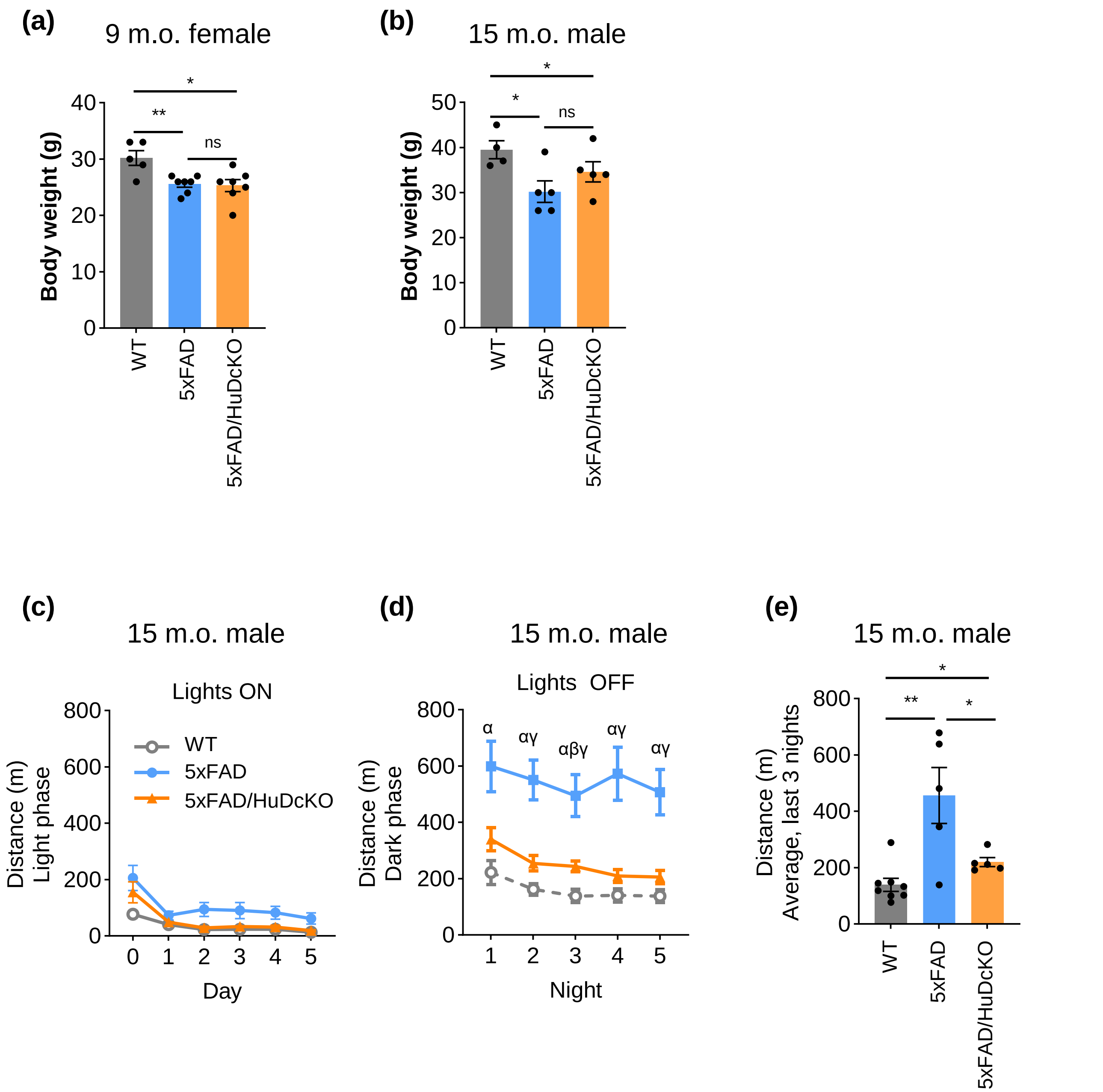

Figure S1. Extended data, HuDcKO suppresses AD traits in the 5xFAD mouse. (a,b) 9-month-old (9 m.o.) female (a) and 15 m.o. male (b) mice were weighed before sacrifice (n=5 for WT, 5xFAD, and 5xFAD/HuDcKO). (c-e) For 15 m.o. male mice, the distance traveled in homecage during 12-hour light (c) and 12-hour dark (d) phases were recorded across five nights, and the average distance traveled per night in the last three nights of testing were calculated after the mice had acclimated to new caging and room (e). Data represent the means ± SEM. Statistical significance (*, $p < 0.05$; **, $p < 0.01$) was assessed with Student's t-test. Greek letters denote significant Tukey's pairwise comparisons between genotypes: α, WT *vs* 5XFAD; β, WT *vs* 5xFAD/HuDcKO; γ, 5xFAD *vs* 5xFAD/HuDcKO.
